# A Novel Technique for Improving Bodily Experience in a Non-operable Super–Super Obesity Case

**DOI:** 10.3389/fpsyg.2016.00837

**Published:** 2016-06-16

**Authors:** Silvia Serino, Federica Scarpina, Anouk Keizer, Elisa Pedroli, Antonios Dakanalis, Gianluca Castelnuovo, Alice Chirico, Margherita Novelli, Santino Gaudio, Giuseppe Riva

**Affiliations:** ^1^Applied Technology for Neuro-Psychology Lab, IRCCS Istituto Auxologico Italiano, MilanItaly; ^2^Department of Psychology, Università Cattolica del Sacro Cuore, MilanItaly; ^3^“Rita Levi Montalcini” Department of Neuroscience, University of Turin, TurinItaly; ^4^Psychology Research Laboratory, IRCCS Istituto Auxologico Italiano, Ospedale San Giuseppe, PiancavalloItaly; ^5^Department of Experimental Psychology, Faculty of Social and Behavioural Sciences, Utrecht University, UtrechtNetherlands; ^6^Department of Brain and Behavioral Sciences, University of Pavia, PaviaItaly; ^7^Department of Surgery and Interdisciplinary Medicine, University of Milano-Bicocca, MilanItaly; ^8^Centre for Integrated Research, Area of Diagnostic Imaging, University Campus Bio-Medico, RomeItaly; ^9^Department of Neuroscience, Functional Pharmacology, Uppsala University, UppsalaSweden

**Keywords:** super–super obesity, virtual reality, body dissatisfaction, body-size distortions, bodily illusion

## Abstract

**Introduction:** The available clinical guidelines for super-super obese patients (i.e., with body mass index (BMI) > 60 kg/m^2^) that are not suitable for bariatric surgery mandate a palliative multidisciplinary treatment (i.e., production and maintenance of weight loss) provided in a center of excellence. However, the modality and the impact of this approach are still controversial. Moreover, it is not able to address the high level of body dissatisfaction and body distortions that are common among these patients.

**Clinical Presentation:** We report the case of a non-operable super–super obesity – a 37 year old woman with a BMI of 62 kg/m^2^ – receiving a specialized treatment for her obstructive sleep apnea. She entered a multidisciplinary program that promoted healthy behaviors, including physical activities and psychological intervention. To improve body dissatisfaction, which was linked to a significant multisensory impairment of body perception, she also entered a virtual reality (VR) body-swapping illusion protocol. At the end of the current investigation, the patient continued her multidisciplinary program, reporting an increase in the motivation for undertaking healthy behavior and a decrease in the anxiety feelings associated with her clinical condition.

**Conclusion:** This case provides preliminary evidence that both body dissatisfaction and body-size distortions of non-operable super-super obesity patients could be addressed by a VR body-swapping protocol, which is important because the palliative multidisciplinary treatment recommended for these patients is not able to address them. Interestingly, the use of a VR body-swapping illusion protocol seems to be able to improve not only the experience of the body in these patients but their motivation for change, too.

## Introduction

The most recent available data estimated that 6.4% of the US adults are morbidly obese (Ogden et al., 2014), suggesting that over a million of individuals suffers from super-super obesity (Finkelstein et al., 2012; Somers and Carter, 2015). For obesity, bariatric surgery is currently considered the most effective option and has been the treatment of choice in the last decade (Buchwald and Oien, 2013), but the importance of stand-alone and/or combined treatment options, particularly for obese patients with being eating pathology, has also been heightened in the literature (e.g., Grilo et al., 2011; McElroy et al., 2015). However, bariatric surgery is not a clear treatment option for a significant proportion of super-super obese patients (i.e., those with body mass index (BMI) > 60 kg/m^2^) not suitable for surgical intervention: surgical treatment of these patients is associated with higher incidence of major complications and mortality (Buchwald et al., 2007; Somers and Carter, 2015). The available clinical guidelines for these patients recommend as mandatory a palliative treatment (i.e., diet, physical activity, and behavior modification) provided in a center of excellence (e.g., Somers and Carter, 2015). Yet, the modality and the impact of this approach are still controversial (Somers and Carter, 2015). Moreover, this approach is not able to address the high level of body dissatisfaction (Rosenberger et al., 2006; Ghai et al., 2014) and body-size distortions (Guardia et al., 2013; Scarpina et al., 2014), which are common among these patients and have a significant impact on their quality of life and psychosocial functioning (Schwartz and Brownell, 2004; Docteur et al., 2010).

This report presents a multidisciplinary approach that focuses on the experience of the body. Specifically, we will discuss the use of a virtual reality (VR) body-swapping illusion (see Petkova and Ehrsson, 2008; Slater et al., 2010 and the references therein) as a novel potential technique to improving the body experience (Gutiérrez-Maldonado et al., 2016; Riva et al., 2016) of a non-operable super-super obesity case, supporting the recovery of motivation for change.

## Case Report

The patient was a 37-year-old woman with a BMI of 62.2 kg/m^2^. She was married, with one child, and had been hospitalized for 5 weeks at the IRCCS Istituto Auxologico Italiano –Ospedale San Giuseppe (Italy), receiving a specialized treatment [i.e., continuous positive airway pressure (CPAP) therapy] for her obstructive sleep apnea. Diagnostic procedures [based on the criteria in the Structured Clinical Interview for DSM-IV, Axis I Disorders (American Psychiatric Association [APA], 2000)] revealed the absence of psychiatric (e.g., Dakanalis et al., 2015a,b) or neuroendocrine comorbidities (e.g., Guardia et al., 2013). In parallel to the treatment for her obstructive sleep apnea, given her clinical situation (i.e., she was not suitable for a surgical intervention), the patient was asked to enter into a multidisciplinary program that promoted healthy behaviors, including physical activities and psychological intervention. Specifically, the psychological team would help her to understand psychological factors associated with her obesity and assess different psychological dimensions related to her condition (e.g., motivation, body dissatisfaction, etc.). The patient agreed to enter in this multidisciplinary program and gave her informed consent, and during the first psychological interview, she reported a dramatic and rapid increase in weight after (approximately 1 year) her marriage. Her lifestyle had become extremely sedentary. She reported the presence of occasional episodes of binge eating, accompanied by a strong feeling of distress. The patient’s family support appeared extremely poor since she reported relational difficulties with her husband’s family. On the Binge Eating Scale (BES; Gormally et al., 1982), the patient obtained a score of 14, coherently with the reported sporadic unhealthy episodes of binge eating (i.e., higher scores indicating more binge eating behavior, with a clinical cut-off of 17). On the Body Attitude Test (BAT; Probst et al., 1995; **Table 1**), the patient revealed a tendency to devalue her own body, concurrently with subjectively reported feelings of distress. In general, the patient expressed a strong body dissatisfaction, which included concerns about body shape and lack of familiarity with her own body.

**Table 1:** Results from Body Attitude Test (BAT).

	Score	Range	Item
**BAT^a^**			
Total Score	82	0–100	
**BAT 1**			
Negative appreciation of body size	35	0–35	*“I have a strong desire to be thinner”*
**BAT 2**			
Lack of familiarity with one’s own body	30	0–35	*“My body in threat for me”*
**BAT 3**			
General body dissatisfaction	15	0–20	“*When I look myself in the mirror, I’m dissatisfied with my own body*”

*^a^Two items are grouped in the so-called “rest factor”.*

**FIGURE 1:**
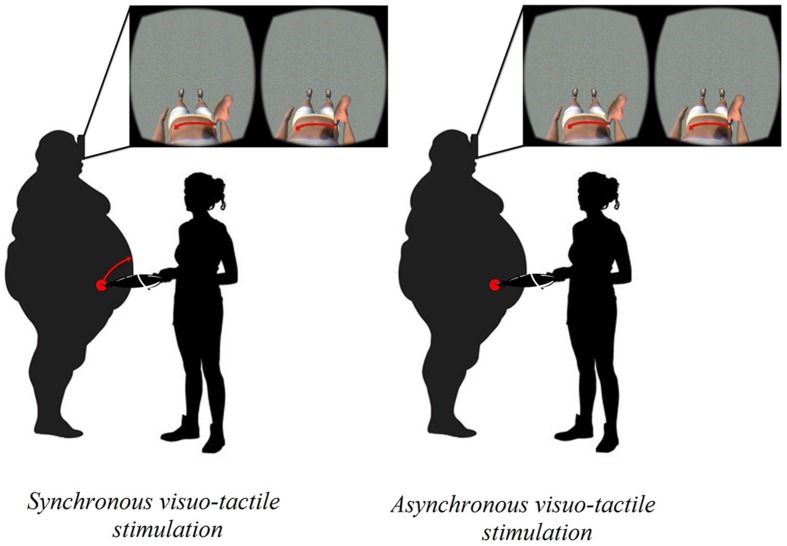
**The VR-body swap illusion**.

**FIGURE 2:**
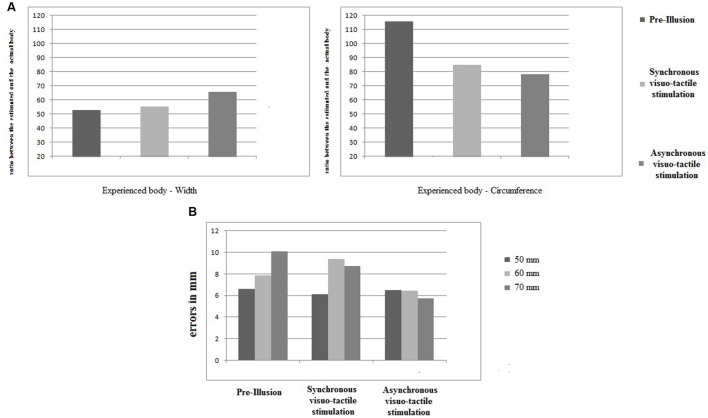
The results relative to body representation tasks. **(A)** Visually based body parts estimation task. For the width (left panel) and the circumference (left panel), the estimation for the three different conditions (“*pre-illusion”*, “*synchronous visuo-tactile stimulation”* and “*asynchronous visuo-tactile stimulation”*) were represented as a ratio between estimated and actual body (*y* axis). **(B)** Tactile-based body parts estimation task. For the three different measures (50 mm left panel; 60 cm middle panel, 70 mm right panel), the error were reported in mm on the *y* axis. Overall, 45 trials were administered: 15 for each measure for each experimental conditions. Eight trials (3 at the “*pre-illusion*”; 2 at the “*synchronous visuo-tactile stimulation*”; 3 at the “*asynchronous visuo-tactile stimulation*”) were excluded since the patient did not perceive the tactile stimulus.

We decided to deeply investigate whether patient would present also an inability to accurately estimate her body size (i.e., body-size distortions), using two tasks involving multiple modalities of body perception, i.e., a visually based body parts estimation task and a tactile-based estimation task^1^. In the visually based body parts estimation task (Serino et al., 2016), the patient was asked to stand in front of a wall and to estimate the width of three different parts of her body (i.e., shoulders, abdomen, and hips) by placing adhesive stickers on the wall representing the estimated distance between the left and the right side of the target body part. Furthermore, she was asked to estimate the circumference of the above-mentioned three parts of her body by placing a piece of rope in a circle/oval on the floor. The actual width and circumference of the body of the participant were also measured. Two average body perception indexes (Slade and Russell, 1973) were calculated for the width and circumference of the body size: Estimated Body Size/Actual Body Size × 100. This index expresses the percentage of similarity with respect to physical body, so that values close to 100 represent an estimated body similar to the physical one. In the *tactile-based estimation task* (Keizer et al., 2011, 2012; Scarpina et al., 2014), patient was blindfolded for the duration of the task and the experimenter simultaneously lightly pressed the two points of a caliper on the patient’s abdomen. The patient was asked to estimate the distance between the two tactile stimuli (i.e., 50, 60, and 70 mm) by varying the separation between the thumb and the index finger of the left hand on a table. The difference between the estimated distance and the real distance was calculated for each trial: a negative error indicates an underestimation of the applied distance, while a positive error indicates an overestimation (Scarpina et al., 2014). The results from these well-validated body representation tasks showed that the patient presented profound body-size distortions. Specifically, she showed underestimation of the width of her body size, and profound overestimation of her body circumference and of tactile distances (see first column in **Figures 2A,B**), suggesting a multisensory impairment of body perception. Since this significant negative bodily experience (i.e., strong body dissatisfaction, which appears to be linked to a significant body-size distortions), we decided to intervene during the first week of her recovery period using the VR body-swapping illusion (Gutiérrez-Maldonado et al., 2016; Riva et al., 2016; Serino et al., 2016; **Figure 1**).

In fact, an increasing amount of work has revealed that embodiment of a virtual body that substitutes for one’s own body by means of a visuo-tactile stimulation (i.e., body-swapping illusion) can lead to altered perceptions of one’s own actual body (e.g., Normand et al., 2011; Piryankova et al., 2014; Serino et al., 2016). Normand et al. (2011) suggested that experiencing the illusory feeling of ownership of a virtual body with a skinny belly may produce changes on body perception, which “could have implications for use in a goal-directed therapy, where patients could, from a first person perspective, experience themselves as being how they want to be as a strong motivator for successful completion of the therapy program”. **Figure 2** shows a comprehensive overview of the results obtained for the body representation tasks.

Concerning estimating the width of the body (**Figure 2A**), the patient initially showed a huge underestimation (i.e., about 50% less than her actual body). After the illusion in both experimental conditions, the tendency towards underestimation persisted. However, an increase in the ratio between estimated and actual measures was observed, indicating that the patients’ body width estimations became more realistic. Concerning estimating the circumference of the body (**Figure 2A**), we initially observed overestimation (i.e., almost 20% greater than the actual body). After the illusion, a decrease in overestimation was observed in both experimental conditions: the ratio dropped to underestimation of the circumference of the body. As concerns tactile estimations (**Figure 2B**), overestimation for 50 mm stimuli remained stable after both the *synchronous* and *asynchronous visuo-tactile stimulation*. The overestimation for the 60 mm stimuli increased after *synchronous visuo-tactile stimulation*, but decreased after the *asynchronous* one. For the 70 mm stimuli, a decrease of the initial overestimation was found for both experimental conditions.

Taking the results from both body representation tasks, we may conclude that: (i) before the illusion was induced, the patient presented profound body-size distortions; (ii) independently from the type of the visuo-tactile stimulation, a modification in the estimated body emerged; and (iii) there was a tendency to reverse the pre-illusion trend, which in turn reduced the contrast observed (i.e., over- and underestimation).

At the end of the current investigation, the patient continued her multidisciplinary program, interestingly reporting an increase in the motivation for undertaking healthy behavior and a decrease in the anxiety feelings associated with her clinical condition. She also reported a better compliance in the continuous positive airway pressure (CPAP) therapy for her obstructive sleep apnea. At the end of her recovery, the patient had lost 5.1 kg (i.e., decrease of 3.7% of her initial weight).

## Conclusion

This case provides preliminary evidence that both body dissatisfaction and body-size distortions of non-operable super-super obesity patients could be addressed by a VR body-swapping protocol, which is important because the palliative multidisciplinary treatment recommended for these patients (Somers and Carter, 2015) is not able to adress them. Specifically, the patient showed a significant body dissatisfaction associated to an underestimation of the width of her body size, and profound overestimation of her body circumference and of tactile distances.

A potential explanation for this multisensory impairment is provided by the Allocentric Lock Theory – ALT (Riva, 2014; Riva et al., 2014, 2015) according to which some obese patients may be locked (i.e., no longer able to update) to an allocentric (observer view) memory of the body that is no longer updated by contrasting egocentric representations driven by perception (Riva, 2014; Riva et al., 2014). In this view, whatever the obese patients can do to modify their real body, they will always be in a “virtual body” that they hate (i.e., body dissatisfaction) and that differs from the real one (i.e., body-size distortions) (Riva et al., 2013, 2014; Riva, 2014; Dakanalis et al., 2016). This is consistent with Guardia et al. (2013), who reported the case of an obese patient experiencing a wider body even after a successful weight reduction.

We evaluated the potential effects of a VR body-swapping illusion on body-size distortions and found that, independent of the type of the visuo-tactile stimulation, the patient showed improvement in estimating her own body size. Although a general tendency persisted towards underestimation of body size, the experience significantly reduced the misalignment (from 110/50 to 70/70) between the visual and the tactile experience of the body. Moreover, as already mentioned at the end of the current investigation, the patient continued her multidisciplinary program, reporting an increase in the motivation for undertaking healthy behavior and a decrease in the anxiety feelings associated with her clinical condition. These findings are particularly relevant since they appear to provide support to the suggestion that a reduction in body-size distortions may motivate extremely obese patients to initiate and maintain healthy behaviors (Normand et al., 2011). However, further exploiting the embodied capabilities of VR (Gutiérrez-Maldonado et al., 2016) in multidisciplinary obesity treatment as means to increase motivation for undertaking healthy behavior by specifically targeting the experience of the body are required.

## Author Contributions

GR and SS developed the study concept. All authors contributed to the study design. SS, FS, EP, AC, GC, and MN were involved in the data collection. SS and AD performed the data analysis and interpretation under the supervision of GR, SS, FS, and AK wrote the first draft of the manuscript. ES, DK, GC, AC, MN, SG, and GR were involved in the critical revision of the manuscript for important intellectual content. All the authors approved the final version of the manuscript for submission.

## Conflict of Interest Statement

The authors declare that the research was conducted in the absence of any commercial or financial relationships that could be construed as a potential conflict of interest.

## Footnotes

1 Traditionally, body experience in obesity population (and eating disorders) has been investigated in terms of perceptual (i.e., inability to accurately estimate body size) or affective (i.e., body dissatisfaction) components (Schwartz and Brownell, 2004; Docteur et al., 2010; Riva et al., 2013; Dakanalis et al., 2014), but experimental evidence highlighting the intuitive direct link between body percept and affective body dissatisfaction exists (see Preston and Ehrsson, 2014 and the references therein)
